# Association between adverse childhood experiences and perinatal depressive symptoms: a cross-sectional analysis of 16,831 women in Iceland

**DOI:** 10.1007/s00737-023-01369-2

**Published:** 2023-09-20

**Authors:** Emma Bränn, Alexandra Vaina, Hilda Björk Daníelsdóttir, Edda Bjork Thordardottir, Qian Yang, Jóhanna Jakobsdóttir, Thor Aspelund, Arna Hauksdóttir, Unnur A. Valdimarsdóttir, Donghao Lu

**Affiliations:** 1 Institute of Environmental Medicine, Unit of Integrative Epidemiology, Karolinska Institutet, Stockholm, Sweden; 2 Centre of Public Health Sciences, Faculty of Medicine, University of Iceland, Reykjavík, Iceland; 3 Mental Health Services, Landspitali, The National University Hospital of Iceland, Reykjavik, Iceland; 4 Department of Medicine, Unit of Clinical Epidemiology, Karolinska Institutet, Stockholm, Sweden

**Keywords:** Pregnancy, Postpartum depression, Childhood maltreatment, Adverse childhood experiences

## Abstract

Emerging data suggest that certain adverse childhood experiences (ACEs) are associated with perinatal depression (PND). However, few studies have comprehensively assessed the cumulative number and types of ACEs and their association to PND. We conducted a cross-sectional analysis among 16,831 female participants from the Stress-And-Gene-Analysis (SAGA) cohort in Iceland, 2018. ACEs were surveyed with the World Health Organization ACE—International questionnaire, while PND symptoms were assessed using the Edinburgh Postnatal Depression Scale (lifetime version). We, while adjusting for confounding factors, estimated the prevalence ratio (PR) of PND in relation to total number of ACEs using the Poisson quasi-likelihood model and further performed analyses for type-specific ACEs. At a mean age of 44 years (SD ± 11.1), 6,201 (36.8%) participants had experienced probable PND. Total number of ACEs was positively associated with PND (PR 1.11 per ACE, 95% CI: 1.10–1.11), also among women without any psychiatric comorbidities (PR 1.13, 95% CI: 1.11–1.14). PRs increased in a dose–response manner with the number of ACEs (P for trend < 0.001); women that endorsed 5 or more ACEs were twice as likely to have experienced PND (PR 2.24, 95% CI: 2.09–2.41). All ACE types (*n* = 13) were associated with PND, with most pronounced association for emotional neglect by a guardian (PR 1.53, 95% CI: 1.47–1.59). Our findings suggest a positive association between number of ACEs and PND symptoms. If our results are confirmed with prospective data, healthcare providers need to be alert of the risk of PND among expecting mothers with history of ACEs.

**Supplementary Information:**

The online version contains supplementary material available at 10.1007/s00737-023-01369-2.

## Introduction

Perinatal depression (PND) affects about 10–20% of pregnant/postpartum women (Woody et al. 2017) with non-negligible influences on both mothers and their families. For instance, women with PND have a higher incidence of preterm delivery, preeclampsia, and suicide; and their children are more likely to have low birth weight, and decreased social, cognitive, and emotional development (Van Niel and Payne 2020). However, few modifiable risk factors have been identified for prevention, except exposure to abusive partners (Gastaldon et al. 2022; Yang K et al. 2022a).

Adverse childhood experiences (ACEs) are traumatic events that occur in childhood (e.g., emotional and physical abuse or neglect and growing up in dysfunctional home environments) and can harm the child’s development and health through changes in the development of the nervous, endocrine, and immune systems under distress (Hughes et al. 2017). Research data support that ACEs might operate through psychobiological pathways leading to PND (Choi and Sikkema 2016). Indeed, previous studies have revealed an association between selected ACEs, e.g., childhood maltreatment, and perinatal mood disorders, especially PND (Osofsky et al. 2021; Racine et al. 2021; Rich-Edwards et al. 2011). However, few studies have made a comprehensive assessment of the different types of adverse events and situations a child can be exposed to (LeMasters et al. 2021; McDonnell and Valentino 2016; Menke et al. 2019; Osofsky et al. 2021). For instance, some subtypes, such as bullying, have not been investigated in relation to PND. Moreover, most studies have focused on any ACEs (Mersky and Janczewski 2018; Miller et al. 2021; Racine et al. 2020; Wajid et al. 2020), and it remains unclear what subtypes of ACEs are most strongly associated with PND. In addition, ACEs are associated with a range of psychiatric disorders in adulthood (Chapman et al. 2004; McKay et al. 2021; Tebeka et al. 2021), and a history of psychiatric disorders predisposes mothers to PND (Guintivano et al. 2018). However, few studies (Tebeka et al. 2021) have taken into account the role of psychiatric comorbidities in the association between ACEs and PND.

Leveraging a large population-representative cohort of females in Iceland, this study aimed to comprehensively examine whether women with any out of 13 different types of ACEs were more likely to experience depressive symptoms during their pregnancy and/or postpartum. We also aimed to identify high-risk subtypes of ACEs most strongly associated to PND and illustrate any risk modification by comorbidity of psychiatric disorders.

## Materials and methods

### Sample characteristics and study design

**Fig. 1:**
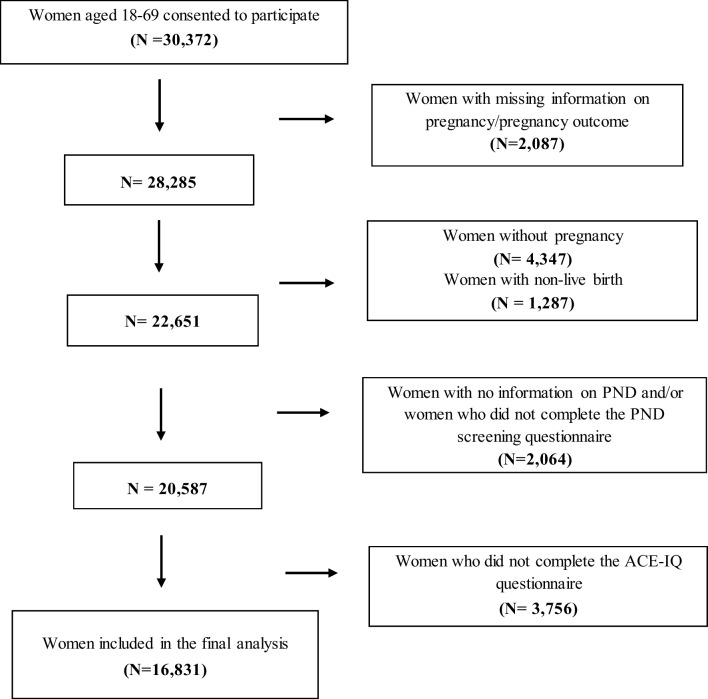
Flowchart of inclusion and exclusion criteria

We conducted a cross-sectional analysis with data from the Stress-And-Gene-Analysis (SAGA) cohort in Iceland, which is representative of the Icelandic female population in terms of demographic characteristics, including age, education, income, and geographic location (Daníelsdóttir et al. 2022). In March 2018, all women aged 18–69 years and living in Iceland were invited to participate in the study by filling in an electronic questionnaire regarding trauma history and current health status. Among 30,372 women who consented to participate (corresponding to approximately 30% of eligible women in Iceland), we identified and included 22,651 women who had given a live birth (Fig. 1). Among them, we excluded 2,064 women due to missing information on PND assessment on more than two items and 3,756 women due to missing information on the ACEs questionnaire. In the end, 16,831 women were included in the analysis (Fig. 1).

### Assessment of PND

Perinatal depressive symptoms were measured using the Edinburgh Perinatal Depression Scale (EPDS) (Cox et al. 1987)—lifetime version (Meltzer-Brody et al. 2013). The EPDS is a ten-item self-reported screening tool for depressive symptoms during pregnancy and/or postpartum and has been proven to have good specificity and sensitivity in other Nordic populations (Eberhard-Gran et al. 2001; SBU 2012). Each item is scored on a scale rangeing from 0 to 3, giving a total score of 30, with higher score reflecting more severe symptoms. The lifetime version of EPDS starts with two screening questions asking if the participant (1) ever felt depressed during pregnancy and (2) ever felt depressed within 6 months after giving birth. Women who answer “yes” to either screening question receive the 10 following questions from the original EPDS to assess the lifetime prevalence of PND, asking for the worst period during pregnancy or after childbirth experienced. For women with missing data on either one or two items, the individual mean value was used for imputation. In accordance with previous studies (Rubertsson et al. 2011), a clinical cut-off of ≥ 13 was used to indicate probable PND. We further divided the women with PND into antenatal, postnatal, and persistent (both antenatal and postnatal) depression according to the first two screening questions, and two additional questions asking for when the symptoms started during pregnancy and when they started postpartum. If depressed both during pregnancy and after childbirth, women were classified as persistent.

### Assessment of ACEs

A modified Icelandic version of the World Health Organization (WHO) ACEs – International questionnaire (IQ) was used to assess ACEs. This widely adopted questionnaire consisted of 39 items on 13 types of ACEs and has been qualitatively tested in six culturally diverse settings (Rutter 2021; WHO 2018). The 13 types included can be grouped into four catergories including abuse (physical, emotional, and sexual abuse), neglect (physical and emotional neglect), household dysfunction (domestic violence, lost of a parent or parental separation, household substance abuse, incarcerated household member, mental illness in the household), and violence (community violence, bullying, and collective violence). Exposure to war or collective violence is extremely rare in Iceland. Consequently, the Icelandic ACE-IQ was modified by adding one screening question on collective violence. As described elsewhere (Daníelsdóttir et al. 2022; Yang Q et al. 2022b), the exposure of each type of ACE was defined according to the Guidance of Analyzing ACE-IQ (frequency version) provided by WHO (WHO 2018). We then calculated the total numbers of ACEs (0–13) for analysis.

### Covariates

Potential confounders included age at time of survey and experience of childhood economic deprivation. To assess the latter, participants were asked “Was your family’s economic situation ever so bad that you suffered any deprivation as a consequence? For example, this could apply to deprivation of nutritious food and/or deprivation of warm clothes and appropriate footwear during the winter months.” We also obtained information on highest educational level, employment status, marital status, and monthly income, surveyed at recruitment, as a proxy for socioeconomic status over the lifetime.

Potential mediators, including parity, age at menarche, and current body mass index (BMI) (derived via self-reported height and weight), were assessed through questions at the recruitment. ACEs has been associated with obesity, whereas pre-pregnancy BMI has been associated with PND (Dachew et al. 2021; Hughes et al. 2017).

Lastly, psychiatric comorbidities including attention deficit hyperactivity disorder (ADHD), autism, schizophrenia, severe depression, bipolar disorder, panic disorder, agoraphobia, social phobia, body dysmorphic disorder, obsessive–compulsive disorder (OCD), specific phobia, general anxiety disorder, adjustment disorder, post-traumatic stress disorder (PTSD), eating disorder (e.g., anorexia nervosa or bulimia nervosa), sleeping disorder, personality disorder, and substance abuse disorder were all collected via self-reports. Current perceived social support was assessed using the Multidimensional Scale of Perceived Social Support (MSPSS), a 12-item questionnaire with scores ranging from 0 to 84, with higher scores suggesting a higher levels of social support. By calculating the mean value of the MSPSS total score, we grouped the participants into low, moderate, and high perceived social support. Categories for all covariates are listed in Table 1.

**Table 1:** Background characteristics of 16,831 Icelandic women with and without PND symptoms, March 2018

	No PND	PND
Total number	10,630 (63.2)	6,201 (36.8)
Age at survey, mean years (± SD)	49.43 (11.22)	44.08 (11.13)
Age at PND, mean years (± SD)	NA	27.82 (5.74)
Childhood deprivation, *n* (%)		
Never	220 (2.1)	420 (6.8)
Seldom	640 (6.0)	706 (11.4)
Sometimes	8,821 (83.0)	4,213 (67.9)
Often	940 (8.8)	855 (13.8)
Unknown	9 (0.1)	7 (0.1)
Age at menarche, years, *n* (%)		
< 10	122 (1.1)	124 (2.0)
10–11	1,697 (16.0)	1,265 (20.4)
12–13	4,926 (46.3)	2,869 (46.3)
14–15	3,316 (31.2)	1,705 (27.5)
≥ 16	563 (5.3)	233 (3.8)
Unknown	6 (0.1)	5 (0.1)
Educational level, *n* (%)		
Primary	1,325 (12.5)	830 (13.4)
Secondary school	2,096 (19.7)	1,308 (21.1)
College or equivalent	3,627 (34.1)	2,157 (34.8)
University degree	2,865 (27.0)	1,481 (23.9)
Unknown	717 (6.7)	425 (6.9)
Parity, *n* (%)		
1	1,870 (17.6)	1,168 (18.8)
2	3,826 (36.0)	2,353 (37.9)
3	3,781 (35.6)	1,927 (31.1)
4	953 (9.0)	616 (9.9)
≥ 5	200 (1.9)	137 (2.2)
Monthly income, ISK, *n* (%)		
< 300,000	2,079 (19.6)	1,755 (28.3)
301,000–500,000	3,164 (29.8)	2,065 (33.3)
501,000–700,000	3,027 (28.5)	1,449 (23.4)
> 701,000	1,951 (18.4)	757 (12.2)
Unknown	409 (3.8)	175 (2.8)
Employment status, *n* (%)		
Working/studying	8,708 (81.9)	4,729 (76.3)
Unemployed/retierd/parental leave	1,871 (17.6)	1,434 (23.1)
Unknown	51 (0.5)	38 (0.6)
Marital status, *n* (%)		
Single/widowed	1,725 (16.2)	1,184 (19.1)
Married/in a relationship	8,885 (83.6)	4,989 (80.5)
Unknown	20 (0.2)	28 (0.5)
BMI, kg/m^2^, *n* (%)		
< 18.5	73 (0.7)	43 (0.7)
18.5–24.9	3,651 (34.3)	1,767 (28.5)
25–29.9	3,673 (34.6)	2,069 (33.4)
> 30	3,010 (28.3)	2,191 (35.3)
Unknown	223 (2.1)	131 (2.1)
Psychiatric comorbidities, *n* (%)		
Depression	574 (5.4)	1,678 (27.1)
Other psychiatric disorders	1,251 (11.8)	1,515 (24.4)
None	8,805 (82.8)	3,008 (48.5)
Perceived social support, *n* (%)		
High	8,729 (82.1)	4,435 (71.5)
Moderate	1,136 (10.7)	1,282 (20.7)
Low	623 (5.9)	364 (5.9)
Unknown	142 (1.3)	120 (1.9)

*PND* perinatal depression, *SD* standard deviation, *ISK* Icelandic Krona, *BMI* body mass index, *MSPSS* Multidimensional Scale of Perceived Social Support. *P* values < 0.001 for all covariates when comparing the distribution between PND and no PND groups using Chi^2^ and *t*-test tests

### Statistical analyses

Prevalence ratios (PR) of PND were estimated in relation to the total number of ACEs by fitting a log-binomial regression through the robust Poisson regression model (Chen et al. 2018). We then analyzed ACEs categorically by comparing individuals who had experienced 1, 2, 3, 4, or ≥ 5 ACE to those without ACEs. To better test the linear assumption, we also visulized the results by the total number of ACEs from 1 to 13.

We constructed four different models for adjustment. In the first model, crude estimates were reported. In the second model, we adjusted for potential confounders, including age and childhood deprivation. In the third model, we additionally adjusted for socioeconomic status at recruitment, including educational level, marital status, employment status, and income. In the final model, the estimates were further adjusted for potential mediators, including age at menarche, BMI, and parity. To reduce tests, we applied the final model in all subsequent association analyses described below.

Similarly, we examined associations for each type of ACEs with PND separately. To understand the independent role of ACE types, the estimates were further adjusted for the other ACEs in the same category.

To investigate potential risk modification by psychiatric comorbidities, we stratified the analyses by the presence of depression, other psychiatric comorbidities, and absence of the psychiatric comorbidities. Moreover, we stratified analyses by age to examine potential recall bias, and by social support to understand the role of current physical and emotional comfort provided by families and friends. To test for effect modification, an interaction term between PND and stratification factor was added and tested for statistical significance as *P* for interaction.

To shed light on PND subtypes, analyses were conducted for antenatal, postnatal, and persistent depression (both antenatal and postnatal depressive symptoms) separately, using all women not included in respective category as reference group. Additionally, we conducted a sensitivity analysis for postnatal depressive symptoms excluding women with postnatal depressive symptoms who experienced premature delivery (< 37 weeks) or serious illness, birth defect, or infant death in the first months (*n* = 356), as these are potentially traumatic events that could account for postnatal depression.

Lastly, to comply with the prevalence of PND in other population settings, a sensitivity analysis was conducted to assess the association of ACEs with PND by using a higher EPDS cut-off score (≥ 22) resulting in a prevalence of 11.1%.

The data were prepared and analyzed through RStudio (Version 1.2.5033) with the statistical significance set at the nominal two-side level of 0.05. This study was approved by the National Bioethics Committee of Iceland (reference number 13–017). Informed consents were obtained from all participants.

## Results

At a mean age of 44 years (standard deviation (SD) 11.1), 6,201 (36.8%) women reported having experienced probable PND. The mean age when they reported suffering PND was 27.8 years (SD 5.7). Compared to women without PND, women with PND were younger at the survey and more likely to have suffered from childhood deprivation and having psychiatric comorbidities including depression. Women with PND were younger at menarche compared to women without PND. Furthermore, at the time of the survey, women who reported PND were more likely to be unemployed, single, obese, as well as to have lower educational levels and monthly income (Table 1).

The prevalence of PND increased from 814 (21.6%) among women without any ACEs to 1,785 (60.2%) among those reporting ≥ 5 ACEs (Table 2). The total number of ACEs was positively associated with PND (Table 2, Model 1: PR 1.14 per ACE, 95% CI 1.13–1.15). After full adjustment, the association was somewhat attenuated yet significant (Table 2, Model 4: PR 1.11 per ACE, 95% CI 1.10–1.11). The PR gradually increased over categories of ACEs. Specifically, women who reported 5 or more ACEs were two times more likely to have experienced PND (Table 2, Model 4: PR 2.24, 95% CI 2.09–2.41). A dose–response relationship was observed between the number of ACEs and PND (*P* for trend < 0.001). The linear relationship was further confirmed when analyzing the number of ACEs from 1 to 13 **(**Online Resource Fig. S1).

**Table 2:** Association between PND and total number of ACEs and ACEs categorized as 0, 1, 2, 3, 4, or ≥ 5 in 16,831 Icelandic women, 2018

	Women, *n*	PND, *n* (%)	Model 1 PR (95% CI)	Model 2 PR (95% CI)	Model 3 PR (95% CI)	Model 4 PR (95% CI)
Total number of ACEs	16,831	6,201 (36.8)	**1.14 (1.13, 1.15)**	**1.12 (1.11, 1.12)**	**1.11 (1.10, 1.12)**	**1.11 (1.10, 1.11)**
By number of ACEs						
0	3,773	814 (21.6)	Ref.	Ref.	Ref.	Ref.
1	3,755	1,082 (28.8)	**1.34 (1.23, 1.45)**	**1.35 (1.25, 1.46)**	**1.33 (1.23, 1.44)**	**1.32 (1.22, 1.42)**
2	2,926	1,032 (35.3)	**1.63 (1.51, 1.77)**	**1.62 (1.50, 1.75)**	**1.59 (1.47, 1.72)**	**1.58 (1.46, 1.70)**
3	2,050	844 (41.2)	**1.91 (1.76, 2.07)**	**1.86 (1.72, 2.01)**	**1.82 (1.68, 1.97)**	**1.79 (1.65, 1.93)**
4	1,361	644 (47.3)	**2.19 (2.02, 2.38)**	**2.02 (1.86, 2.19)**	**1.98 (1.82, 2.14)**	**1.95 (1.79, 2.11)**
≥ 5	2,966	1,785 (60.2)	**2.79 (2.61, 2.98)**	**2.40 (2.23, 2.57)**	**2.30 (2.14, 2.47)**	**2.24 (2.09, 2.41)**

*PND* perinatal depression, *ACE* adverse childhood experience, *PR* prevalence ratio, *CI* confidence interval. Statistically significant values are presented in bold

Model 1: no adjustment

Model 2: adjusted for age at the survey and childhood deprivation

Model 3: additionally adjusted for educational level, marital status, employment status, and income

Model 4: additionally adjusted for BMI, parity, and age at menarche

All individual types of ACEs were positively associated with PND (Table 3). The strongest association was noted for emotional neglect (Table 3, PR 1.53, 95% CI: 1.47–1.59), followed by having someone mentally ill in the household (Table 3, PR 1.48, 95% CI: 1.42–1.54) and emotional abuse by a parent/guardian (Table 3, PR 1.46, 95% CI: 1.40–1.53). When additionally adjusting for ACEs in the same category, associations of loss of a parent or experience of parental separation and incarcerated household member attenuated to null.

**Table 3:** Association between PND and different ACE sub-types, in 16,831 Icelandic women, 2018

ACE	Women, *n*	PND, *n* (%)	PR (95% CI)^a^	PR (95% CI)^b^
Abuse				
Physical				
No	15,985	5,672 (35.5)	Ref.	Ref.
Yes	846	529 (62.5)	**1.38 (1.30, 1.47)**	**1.09 (1.03, 1.17)**
Emotional				
No	14,442	4,759 (33.0)	Ref.	Ref.
Yes	2,389	1,442 (60.4)	**1.46 (1.40, 1.53)**	**1.38 (1.31, 1.44)**
Sexual				
No	11,502	3,762 (32.7)	Ref.	Ref.
Yes	5,329	2,439 (45.8)	**1.28 (1.23, 1.33)**	**1.22 (1.18, 1.27)**
Neglect				
Physical				
No	15,556	5,495 (35.3)	Ref.	Ref.
Yes	1,275	706 (55.4)	**1.23 (1.16, 1.30)**	**1.09 (1.03, 1.15)**
Emotional				
No	11,432	3,412 (29.8)	Ref.	Ref.
Yes	5,399	2,789 (51.7)	**1.53 (1.47, 1.59)**	**1.52 (1.45, 1.58)**
Household dysfunction				
Domestic violence				
No	12,787	4,100 (32.1)	Ref.	Ref.
Yes	4,044	2,101 (52.0)	**1.37 (1.32, 1.43)**	**1.22 (1.17, 1.28)**
Lost a parent or parental separation				
No	10,442	3,580 (34.3)	Ref.	Ref.
Yes	6,389	2,621 (41.0)	**1.06 (1.02, 1.10)**	0.98 (0.94, 1.02)
Household substance abuse				
No	11,289	3,713 (32.9)	Ref.	Ref.
Yes	5,542	2,488 (44.9)	**1.19 (1.15, 1.24)**	**1.05 (1.01, 1.10)**
Incarcerated household member				
No	16,018	5,767 (36.0)	Ref.	Ref.
Yes	813	434 (53.4)	**1.14 (1.07, 1.22)**	0.97 (0.90, 1.03)
Mental illness in the household				
No	11,971	3,592 (30.0)	Ref.	Ref.
Yes	4,860	2,609 (53.7)	**1.48 (1.42, 1.54)**	**1.38 (1.33, 1.44)**
Violence				
Community violence				
No	16,227	5,837 (36.0)	Ref.	Ref.
Yes	604	364 (60.3)	**1.30 (1.22, 1.39)**	**1.24 (1.16, 1.33)**
Bullying				
No	14,393	4,806 (33.4)	Ref.	Ref.
Yes	2,438	1,395 (57.2)	**1.36 (1.31, 1.43)**	**1.35 (1.29, 1.41)**
Collective violence				
No	16,227	5,837 (36.0)	Ref.	Ref.
Yes	604	364 (60.3)	**1.30 (1.22, 1.39)**	**1.24 (1.16, 1.33)**

*PND* perinatal depression, *ACE* adverse childhood experience, *PR* prevalence ratio, *CI* confidence interval. Statistically significant values are presented in bold

^a^Adjusted for age at survey, childhood deprivation, educational level, marital status, employment status, monthly income, BMI, parity, and age of menarche

^b^Additionally adjusted for all the other ACEs in same category, except for violence where community and collective were only adjusted for bullying

In the stratified analysis, a stronger positive association was found among individuals without any psychiatric comorbidities (*P* for interaction < 0.001; Table 4). Moreover, we found a positive association between ACEs and PND across all age groups, although the PR was somewhat greater with increased age (*P* for interaction < 0.001; Table 4). Further, current perceived social support did not clearly modify the association although a weaker PR was noted among women having moderate social support (*P* for interaction < 0.001; Table 4).

**Table 4:** Association between PND and total number of ACEs stratified by comorbidity of psychiatric disorders, age at time of survey, and social support at time of survey, in 16,831 Icelandic women, 2018

	Women, *n*	PND, *n* (%)	PR (95% CI)^a^	*P* for interaction
By comorbidity of psychiatric disorders				< 0.001
Depression	2,252	1,678 (74.5)	**1.01 (1.00, 1.02)**	
Other	2,766	1,515 (54.8)	**1.05 (1.03, 1.06)**	
No	11,813	3,008 (25.5)	**1.13 (1.12, 1.15)**	
By age at time of survey, years				< 0.001
18–29	1,038	575 (55.4)	**1.09 (1.07, 1.11)**	
30–39	3,624	1,776 (49.0)	**1.09 (1.07, 1.10)**	
40–49	4,592	1,871 (40.7)	**1.10 (1.09, 1.11)**	
50–69	7,577	1,979 (26.1)	**1.15 (1.14, 1.16)**	
By social support at time of survey				< 0.001
High support	13,164	4,435 (33.7)	**1.11 (1.10, 1.12)**	
Moderate support	2,418	1,282 (53.0)	**1.05 (1.05, 1.07)**	
Low support	987	364 (36.9)	**1.10 (1.07, 1.13)**	

^a^Adjusted for age at survey, childhood deprivation, educational level, marital status, employment status, monthly income, BMI, parity, and age of menarche. Statistically significant values are presented in bold

In the analysis of PND subtypes, the strongest association was found for persistent depression (both antenatal and postnatal depressive symptoms) (PR: 1.12, 95% CI 1.11–1.13; Table 5). Women that endorsed 5 or more ACEs were almost three times more likely to have experienced persistent PND (PR: 2.83, 95% CI 2.50–3.20; Table 5). The association for postnatal depressive symptoms was comparable in the sensitivity analysis where we excluded women with postnatal depressive symptoms who experienced premature delivery, serious illness, birth defect, or infant death (Online Resource Table S1).

**Table 5:** Association between PND subtypes (women with PND who did not specify in the first two screening questions if the depression occurred during pregnancy or after the delivery excluded (*n* = 173)) and total number of ACEs and ACEs categorized as 0, 1, 2, 3, 4, or ≥ 5 in 16,658 Icelandic women, 2018

		Antenatal				Postnatal				Persistent			
	Women, *N*	AND, *n*	(%)	PR	(95% CI)^a^	PPD, *n*	(%)	PR	(95% CI)^a^	PD, *n*	(%)	PR	(95% CI)^a^
Total number of ACEs	16,658	630	(3.8)	**1.09**	**(1.05, 1.13)**	2,201	(13.2)	**1.09**	**(1.07, 1.11)**	3,197	(19.2)	**1.12**	**(1.11, 1.13)**
By number of ACEs													
0	3,743	101	(2.7)	Ref		363	(9.7)	Ref		320	(8.5)	Ref	
1	3,730	110	(2.9)	1.11	(0.85, 1.45)	432	(11.6)	**1.22**	**(1.07, 1.39)**	515	(13.8)	**1.55**	**(1.36, 1.76)**
2	2,904	104	(3.6)	**1.34**	**(1.02, 1.75)**	413	(14.2)	**1.51**	**(1.32, 1.72)**	493	(17.0)	**1.82**	**(1.60, 2.07)**
3	2,023	94	(4.6)	**1.74**	**(1.31, 2.30)**	279	(13.8)	**1.47**	**(1.27, 1.71)**	444	(21.9)	**2.20**	**(1.93, 2.51)**
4	1,341	65	(4.8)	**1.72**	**(1.26, 2.35)**	200	(14.9)	**1.59**	**(1.34, 1.87)**	359	(26.8)	**2.47**	**(2.15, 2.83)**
≥ 5	2,917	156	(5.3)	**1.75**	**(1.33, 2.29)**	514	(17.6)	**1.92**	**(1.67, 2.20)**	1,066	(36.5)	**2.83**	**(2.50, 3.20)**

*AND* antenatal depression, *PPD* postnatal depression, *PD* persistent depression (both antenatal and postnatal), *ACE* adverse childhood experience, *PR* prevalence ratio, *CI* confidence interval. Statistically significant values are presented in bold

^a^Adjusted for age at survey, childhood deprivation, educational level, marital status, employment status, monthly income, BMI, parity, and age of menarche

Last, in a sensitivity analysis of using a higher threshold for PND classification (EPDS cut-off score ≥ 22; prevalence 11.1%), an even stronger association was noted between the total number of ACEs and PND (PR: 1.18, 95% CI 1.16–1.20; Online Resource Table S2).

## Discussion and conclusions

In this nationwide study of 16,831 Icelandic women with population representative sociodemographic characteristics, the findings indicated a positive association between the accumulative number of ACEs and PND symptoms in a dose–response manner. Specifically, women who were exposed to ≥ 5 ACEs had a twofold higher risk of PND than women with no ACEs. Moreover, we found that all 13 studied types of ACEs were positively associated with PND, albeit to a varying extent. The strongest association was noted for emotional neglect, followed by having someone mentally ill in the household and emotional abuse during childhood. While studies suggest impaired emotional clarity and regualtion as a possible mechanism linking emotional neglect to major depression (Jessar et al. 2017), further research is needed to understand why these ACEs are particularly associated with PND. Notably, in contradiction to most of the other ACEs where anyone can be considered the offender, emotional neglect and emotional abuse are specifically asked as the parent being the one that neglected or abused. Losing a parent or parental separation and having a household member incarcerated did not increase the risk of PND when other household dysfunctions were accounted for. In addition, we observed a stronger association between ACEs and PND among individuals without a comorbidity of psychiatric disorders. The association was most pronounced in women reporting persistent (both antenatal and postnatal) perinatal depression.

Although the SAGA cohort is representative of the Icelandic female population (Daníelsdóttir et al. 2022), the prevalence of PND in this study was 36.7%, which is considerably higher than in other studies (PR: 11.9–17%) (Shorey et al. 2018; Woody et al. 2017). The use of the lifetime version of the EPDS in our study may contribute to this higher prevalence as it will present a cumulative prevalence of PND from multiple pregnancies. Previous studies, although with dissimilar settings, have reported both higher (Kiewa et al. 2022; Meltzer-Brody et al. 2013) and lower prevalence (Viktorin et al. 2016) of PND using the EPDS lifetime version. In a sensitivity analysis where we increased the EPDS cut-off threshold to 22, the prevalence of PND was more comparable to other population settings (Woody et al. 2017), and we found an even stronger association between ACEs and PND symptoms.

Our results of accumulative number of ACEs and risk of PND are in line with previous studies on a single or fewer types of ACEs (Chung et al. 2008; Kumar et al. 2018; Leeners et al. 2014; Li et al. 2017; Osofsky et al. 2021; Racine et al. 2020; Rich-Edwards et al. 2011; Robertson-Blackmore et al. 2013; Samia et al. 2020; Tebeka et al. 2021; Zhang et al. 2019). A few studies measuring multiple types of ACEs have also indicated a does-response relationship (Tebeka et al. 2021; Wajid et al. 2020). However, none of these studies covered 13 ACE subtypes in one sample and analysed associations of each of them with PND. Particularly those not included in the pioneer study by Felitti et al. (1998) (e.g., loss of parent or parental separation, community and collective violence and bullying) have been less studied although some are common adverse experiences in childhood (Modecki et al. 2014). In line with previous studies, we also found positive associations of reported physical and emotional neglect (Lang et al. 2006; Li et al. 2017; Tebeka et al. 2021), emotional, sexual, and physical abuse (Benedict et al. 1999; Leeners et al. 2014; Robertson-Blackmore et al. 2013; Tebeka et al. 2021), and household dysfunction (Osofsky et al. 2021) during childhood and PND symptoms. Domestic violence was positively associated with PND in our study and has previously been associated with depressive symptoms at 36 months postpartum (LeMasters et al. 2021), while other studies (Chung et al. 2008) found domestic violence not significantly associated with antenatal depression, possibly due to different assesment of PND. Most importantly, being bullied during childhood has been associated with mental ill-health outside the perinatal period (McKay et al. 2021). However, bullying has not been analyzed separately in relation to PND although included in a couple of studies (LeMasters et al. 2021; Samia et al. 2020). Our findings indeed indicate that bullying is associated with an increased risk of PND symptoms.

Both ACEs (Copeland et al. 2018) and PND (Yang K et al. 2022a) have been associated with lower educational attainment, risky behaviours, and social functioning. In our study, the estimates were only slightly reduced when adjusting for educational level, income, and employment status at survey, indicating that these characteristics do not contribute significantly to the noted association between ACEs and PND.

ACEs are associated with an increased risk of a range of psychiatric disorders, including depression (Chapman et al. 2004; McKay et al. 2021; Tebeka et al. 2021). Moreover, it has been well recognized that pre-existing psychiatric disorders, especially depressive disorders, constitute an important risk factor of PND (Gastaldon et al. 2022; Guintivano et al. 2018; Howard et al. 2014; Yang K et al. 2022a). It is therefore plausible that pre-existing psychiatric disorders mediate the observed association between ACEs and PND. However, we found a positive association between ACEs and PND among individuals with and without psychiatric comorbidities, respectively. This suggests that pre-existing psychiatric disorders cannot completely explain our findings on the association between ACEs and PND symptoms.

Maltreatment during childhood is associated with morphological alterations in the brain (Teicher and Samson 2016), and ACEs might operate through psychobiological pathways leading to PND (Choi and Sikkema 2016). After exposure to a threatening event in childhood, the hypothalamic-pituitary-adrenal (HPA) axis is activated causing an increase in corticosteroids, which interact with cognitive and physical functions (de Kloet et al. 1999). Young children are more vulnerable to overexposure to corticosteroids induced by regular stress events and are therefore predisposed for stress-related disorders in adulthood (Chapman et al. 2004; Suzuki et al. 2014). Notably, during pregnancy, the placenta produces corticotrophins releasing hormone, activating the pituitary gland which in turn activates the adrenal gland to produce corticosteroids. Indeed, ACEs have been associated with heightened HPA axis activity and higher cortisol levels during pregnancy (Schreier et al. 2015). Moreover, the association between having someone mentally ill in the household during childhood and PND suggests potential confounding by familial factors, such as shared genetics between the participant with PND and the family member who was mentally ill. Further, psychosocial factors such as low social and emotional support by family members (Cho et al. 2022; Racine et al. 2020) and low resilience to distress in adulthood in women experiencing ACEs (Daníelsdóttir et al. 2022) could contribute to the association between ACEs and PND. Future studies may also assess other factors, such as adverse events in adulthood and comorbidities such as premenstrual syndrome, which perhaps could mediate the observed association.

### Strengths and limitations

The major strength of this study is the large sample size, which allows us to explore the association of multiple ACEs and both PND subtypes and ACE subtypes. Moreover, our sample is representative of the Icelandic female population (Daníelsdóttir et al. 2022), whereas many previous studies on this association are based on smaller samples from clinical settings (Li et al. 2017; Samia et al. 2020; Tebeka et al. 2021).

One limitation of this study is the nature of the cross-sectional setting, which precludes any inferences on causality. In our study, the timeline between the exposure and outcome seems established, although both data were collected retrospectively. Moreover, there might be some misclassification of ACEs. The participants had to recall experiences that occurred during childhood at an average age of 44 years. However, we found a robust association between ACEs and PND among individuals aged 18–29 years, reducing concerns of recall bias. In addition, a validity study has found a good agreement between ACEs and the recall in adulthood (Dube et al. 2004). Mental state, such as ongoing symptoms of depression, can affect how childhood memories are viewed and recalled (Köhler et al. 2015). Although we found a strong association in women without psychiatric disorders, subclinical conditions could still result in recall bias on ACEs.

Furthermore, we might have misclassified some PND cases. The EPDS is designed as a screening tool for PND. To our knowledge, the EPDS has not been validated in the Icelandic population, although it has a high specificity and sensitivity in other Nordic countries, e.g. Sweden (specificity 88% and sensitivity 72%) (SBU 2012). Women who have experienced ACEs are more likely to report psychological problems, including PND. Hence, it is possible that the misclassification of PND is differential. However, the observed association between PND and ACEs is even stronger in the absence of psychiatric comorbidities, which decreases this possibility. Moreover, we used a modified EPDS (which captures lifetime prevalence), where women are asked to recall their experiences during and after pregnancy. Recall bias is therefore also a possibility, although it has been shown that the lifetime version is reliable enough for epidemiological studies (Meltzer-Brody et al. 2013). As mentioned above, the robust association noted in the group aged 18–29 somewhat alleviates the concern of potential recall bias in PND. Further, due to the structure of how data was collected, we were unable to assess when in relation to the delivery the EPDS was completed. In addition, we cannot rule out the possibility of residual confounding.

### Clinical application

The results of this study suggest that screening pregnant women for ACEs could help identify women vulnerable to PND. Early identification of high-risk individuals may aid early detection and potential intervention, which would not only reduce the burden for the mother and her baby (O’Connor et al. 2019), but also the cost to the society (Bauer et al. 2016).

## Conclusions

Our findings suggest that ACEs are positively associated with perinatal depressive symptoms in a dose–response manner, with emotional neglect, having someone mentally ill in the household, and emotional abuse by a parent/guardian presenting the strongest association. Our findings highlight the potential utility of screening for ACEs at maternal healthcare and may call for targeted prevention strategies for women with ACEs to lower the risk of PND. Finally, it is of utmost importance to prevent ACEs among children worldwide to minimize the impact on their adult life and intergenerational transmission of adverse health effects.

### Supplementary Information

ESM 1 (DOCX 28.6 KB)

## Data Availability

The datasets used and/or analysed during the current study are available from the corresponding author on reasonable request.
