# Supplementary material for: Association between adverse childhood experiences and perinatal depressive symptoms: a cross-sectional analysis of 16,831 women in Iceland
Source: Arch Womens Ment Health. 2023 Sep 20;26(6):839–49. doi: 10.1007/s00737-023-01369-2 (PMC10632282; doi:10.1007/s00737-023-01369-2)
Supplement: Supplementary file 1 — (DOCX 28.6 KB) [file 737_2023_1369_MOESM1_ESM.docx]

**Supplementary Information**

Association between adverse childhood experiences and perinatal depressive symptoms: a cross-sectional analysis of 16,831 women in Iceland

Authors: Emma Bränn^1^ and Alexandra Vaina^1^*, Hilda Björk Daníelsdóttir^2^, Edda Bjork Thordardottir^2,3^, Qian Yang^4^, Jóhanna Jakobsdóttir^2^, Thor Aspelund^2^, Arna Hauksdóttir^2^, Unnur A. Valdimarsdóttir^1,2^, Donghao Lu^1^

*Shared first authorship

Affiliations:

1. Institute of Environmental Medicine, Unit of Integrative Epidemiology, Karolinska Institutet, Sweden
2. Centre of Public Health Sciences, Faculty of Medicine, University of Iceland, Reykjavík, Iceland
3. Mental Health Services, Landspitali, The National University Hospital of Iceland, Reykjavik, Iceland
4. Department of Medicine, Unit of Clinical Epidemiology, Karolinska Institutet, Sweden

Corresponding author: Emma Bränn ([emma.brann@ki.se](mailto:emma.brann@ki.se)), Karolinska Institutet, Institute of Environmental Medicine, ORCID: 0000‐0001‐9664‐7973

**Online Resource Tabel S1.** Association between postnatal depressive symptoms and total number of ACEs and ACEs categorized as 0, 1, 2, 3, 4 or ≥5, excluding women with postnatal depressive symptoms who experienced premature delivery (<37 weeks) or serious illness, birth defect or infant death.

|  | **Women, n** | **PPD, n(%)** | **PR ^a.^** | **95% CI** | |
| --- | --- | --- | --- | --- | --- |
| **Total number of ACEs** | 16,302 | 1,845 (11.3) | **1.10** | **(1.08,** | **1.12)** |
| **By number of ACEs** |  |  |  |  |  |
| 0 | 3,685 | 305 (8.3) | Ref. | | |
| 1 | 3,656 | 358 (9.8) | **1.22** | **(1.06,** | **1.41)** |
| 2 | 2,846 | 355 (12.5) | **1.58** | **(1.36,** | **1.82)** |
| 3 | 1,980 | 236 (11.9) | **1.52** | **(1.29,** | **1.79)** |
| 4 | 1,310 | 169 (12.9) | **1.65** | **(1.38,** | **1.97)** |
| ≥5 | 2,825 | 422 (14.9) | **1.99** | **(1.71,** | **2.31)** |
| PPD = Postnatal depression, ACE = Adverse childhood experience, PR = Prevalence ratio, CI = confidence interval. | | | | | |
| a.  adjusted for age at survey, childhood deprivation, educational level, marital status, employment status, monthly income, BMI, parity, and age of menarche | | | | | |
| *Women with PND who did not specify in the first two screening questions if the depression occurred during pregnancy or after the delivery excluded (n= 173). | | | | | |

**Online Resource Table S2**. Association between PND, using EPDS cut-off score ≥22, and total number of ACEs and ACEs categorized as 0, 1, 2, 3, 4 or ≥5.

|  | **Women, n** | **PND, n (%)** | **Model 1, PR (95% CI)** | **Model 2, PR (95% CI)** | **Model 3, PR (95% CI)** | **Model 4, PR**  **(95% CI)** |
| --- | --- | --- | --- | --- | --- | --- |
| **Total** | 16,831 | 1,875 (11.1) | 1.23 (1.22, 1.25) | 1.19 (1.17, 1.21) | 1.18 (1.16, 1.20) | 1.18 (1.16, 1.20) |
| **number of ACEs** |  |  |  |  |  |  |
| **By number of ACEs** |  |  |  |  |  |  |
| 0 | 3,773 | 154 (4.1) | Ref. | Ref. | Ref. | Ref. |
| 1 | 3,755 | 262 (7.0) | **1.71 (1.41, 2.08)** | **1.75 (1.45, 2.12)** | **1.72 (1.42, 2.08)** | **1.69 (1.40, 2.05)** |
| 2 | 2,926 | 275 (9.4) | **2.30 (1.90, 2.79)** | **2.29 (1.90, 2.77)** | **2.23 (1.84, 2.69)** | **2.19 (1.81, 2.65)** |
| 3 | 2,050 | 234 (11.4) | **2.80 (2.30, 3.40)** | **2.72 (2.24, 3.31)** | **2.62 (2.15, 3.18)** | **2.53 (2.08, 3.08)** |
| 4 | 1,361 | 224 (16.5) | **4.03 (3.32, 4.90)** | **3.56 (2.92, 4.34)** | **3.43 (2.81, 4.18)** | **3.35 (2.75, 4.09)** |
| ≥5 | 2,966 | 726 (24.5) | **6.00 (5.07, 7.09)** | **4.77 (4.00, 5.69)** | **4.42 (3.70, 5.28)** | **4.27 (3.57, 5.10)** |
| PND = Perinatal depression, ACE = Adverse childhood experience, PR = Prevalence ratio, CI = confidence interval.  Model 1: No adjustment.  Model 2: adjusted for age at the survey and childhood deprivation.  Model 3: additionally adjusted for educational level, marital status, employment status, and income.  Model 4: additionally adjusted for BMI, parity, and age at menarche | | | | | | |

**Online Resource Figure S1**. Association between PND and number of ACEs categorical from 0 to 13 displayed as prevalence ratio with 95% CI. Estimates adjusted for age at survey, childhood deprivation, educational level, marital status, employment status, monthly income, BMI, parity, and age of menarche.
